# Supplementary material for: The Influence of the Genotype and Planting Density on the Structure and Composition of Root and Rhizosphere Microbial Communities in Maize
Source: Microorganisms. 2023 Sep 28;11(10):2443. doi: 10.3390/microorganisms11102443 (PMC10608840; doi:10.3390/microorganisms11102443)

Supplementary Figure S1. The shoot architecture of two maize accessions used in this study. This figure shows the growth performance and shoot architecture of maize Guangliangtian27（G27） and Mintian6855(M6855) under the normal field conditions. Bars = 20 cm.

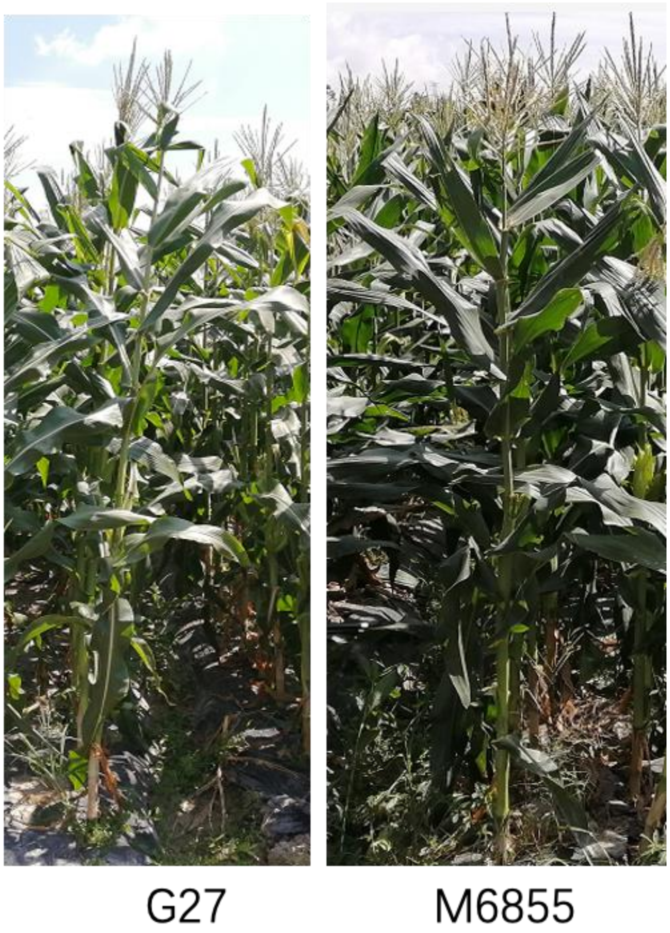

Supplementary Figure S2. The diagram of plant cultivation under different density conditions. In each plot 8 rows of plants were designed with 60 cm row space. The size of plot is 500 cm * 480 cm. In the low-density plot (left) the plant distance is 36 cm, while in the high-density plot (right) the plant distance is 24 cm.

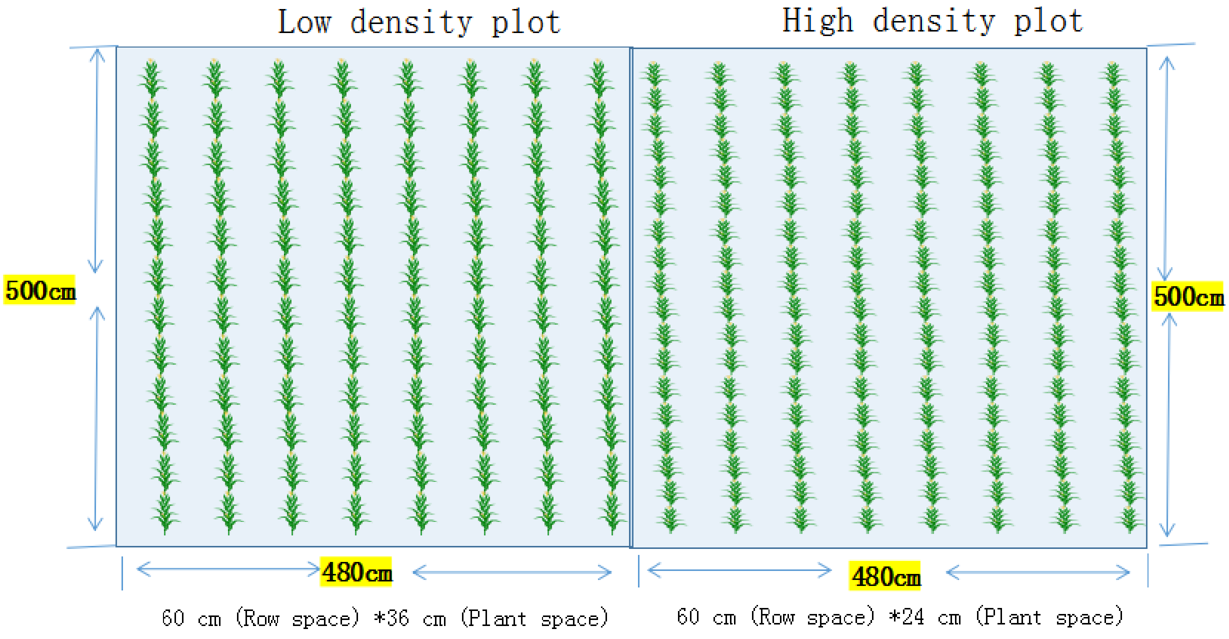

Supplementary Figure S3. The diagram of plant cultivation under different density conditions.

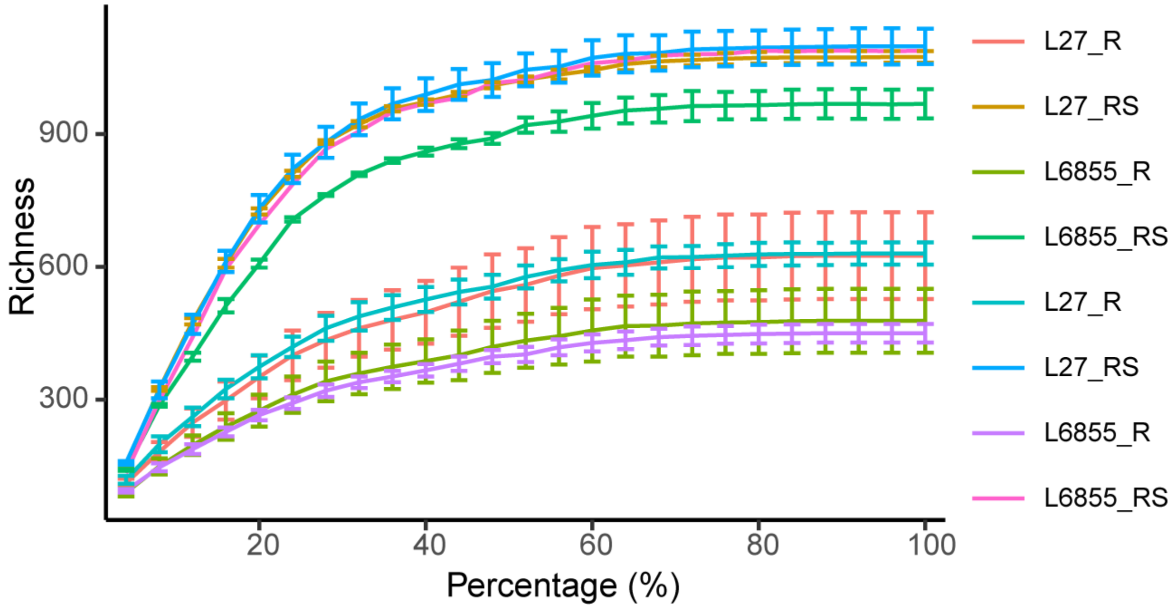

Supplementary Figure S4. The summary of the sequencing depth.

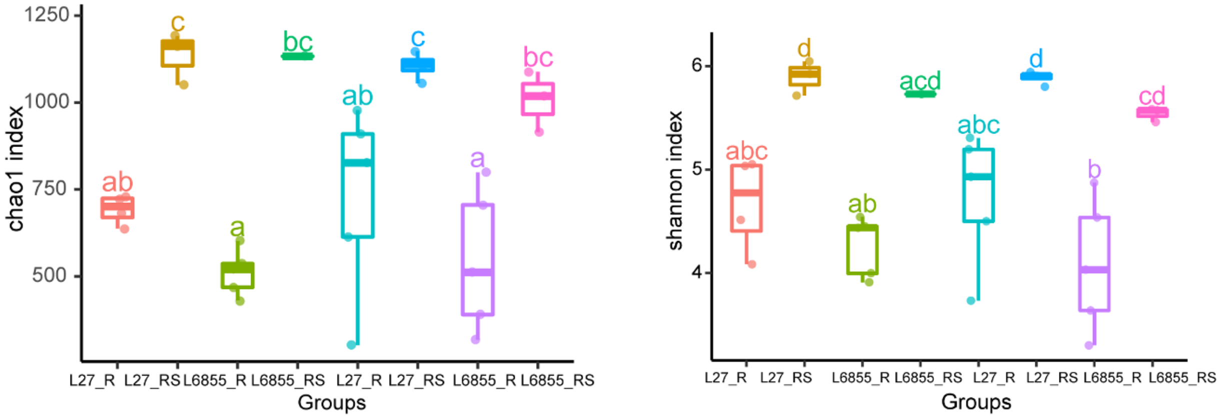

Supplement: Supplementary file 1 [file microorganisms-11-02443-s001.zip › microorganisms-2507171-supplementary.pdf]
